# CNS demyelination associated with immune dysregulation and a novel *CTLA-4* variant

**DOI:** 10.1177/1352458520963896

**Published:** 2021-06-07

**Authors:** Stefania Kaninia, Alexandros Grammatikos, Kathryn Urankar, Shelley A Renowden, Nikunj K Patel, Mark M Gompels, Claire M Rice

**Affiliations:** Clinical Neurosciences, Translational Health Sciences, Bristol Medical School, University of Bristol, Bristol, UK/North Bristol NHS Trust, Bristol, UK; North Bristol NHS Trust, Bristol, UK; North Bristol NHS Trust, Bristol, UK; North Bristol NHS Trust, Bristol, UK; North Bristol NHS Trust, Bristol, UK; North Bristol NHS Trust, Bristol, UK; Clinical Neurosciences, Translational Health Sciences, Bristol Medical School, University of Bristol, Bristol, UK/North Bristol NHS Trust, Bristol, UK

**Keywords:** CTLA-4, demyelination

## Abstract

**Background:**

The cytotoxic T-lymphocyte antigen-4 (CTLA-4) pathway acts as a negative immune regulator of T-cell activation and promotes self-tolerance.

**Case:**

We report the first case of biopsy-proven central nervous system inflammatory demyelination in the context of primary immunodeficiency and a novel *CTLA-4* variant.

**Conclusion:**

This case has significant implications for the development of novel treatments for autoimmune conditions including multiple sclerosis and further emphasises the need for caution with clinical use of CTLA-4 immune checkpoint inhibitors in those with a history of inflammatory demyelination.

## Introduction

Cytotoxic T-lymphocyte antigen-4 (CTLA-4) is CD28 homologue which negatively regulates T-cell activation and promotes tolerance; in contrast to CD28, CTLA-4 binding fails to stimulate T cells. Indeed, *CTLA-4* variants are recognised to cause immune dysregulation disorders and CTLA-4 blockade is employed in cancer therapy. However, the use of CTLA-4 checkpoint inhibitors has been complicated by the emergence of autoimmunity, including demyelination.^1^ We report the first case of biopsy-proven central nervous system (CNS) inflammatory demyelination in the context of primary immunodeficiency and a novel *CTLA-4* variant.

## Case report

A 51-year-old woman presented with a 3-week history of poor balance, nausea and right-sided facial sensory disturbance. Her medical history was significant for bronchiectasis, necrotising granulomatous lymphadenitis, iron deficiency anaemia, eczema, idiopathic thrombocytopenic purpura, recurrent sinusitis, osteonecrosis of the jaw induced by bisphosphonates, diverticulitis, bowel salt malabsorption and vitamin D deficiency. The patient’s daughter had recurrent episodes of CNS inflammation as a child and in adulthood she developed autoimmune hepatitis, autoimmune haemolytic anaemia and bronchiectasis. The patient’s son was known to have type I diabetes, thyroid disease, pernicious anaemia and autoimmune encephalitis. *FAS* sequencing for autoimmune lymphoproliferative syndrome (ALPS) was normal and, at the time of presentation, extended panel screening for primary immunodeficiency was ongoing. There was prior exposure to corticosteroids but no other immunomodulatory treatment.

**Figure 1.**
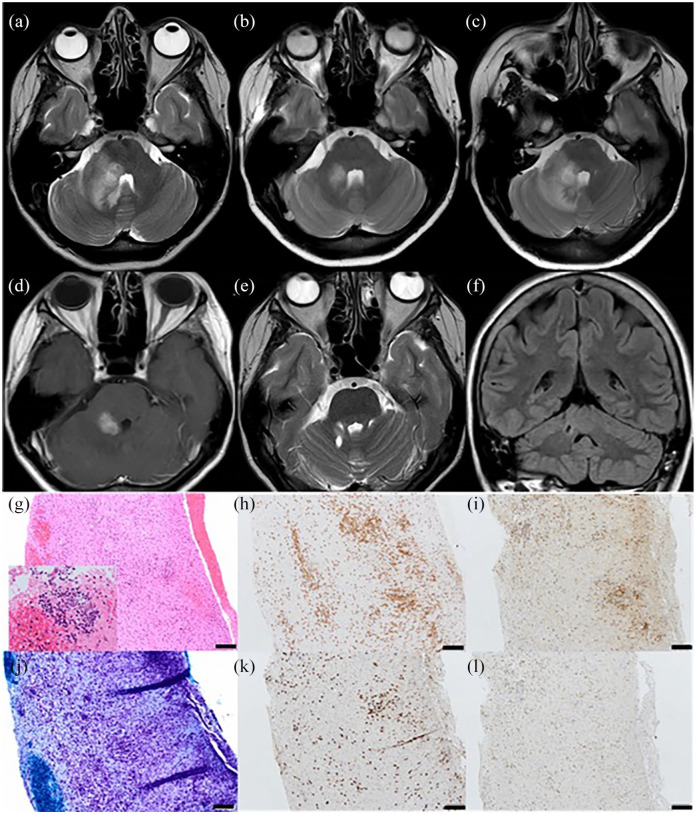
Neuroimaging: (a–c, e) T2 axial MRI brain, (d) gadolinium-enhanced T1 axial MRI brain and (f) fluid-attenuated inversion recovery (FLAIR) coronal MRI brain. (g–l) Histology of cerebellar white matter biopsy. Neuroimaging at presentation (a) demonstrated an intrinsic lesion in the right cerebellar peduncle with surrounding oedema which improved with corticosteroids (b) but relapsed on steroid wean (c). The intrinsic cerebellar lesion demonstrated nodular enhancement (d). Follow-up neuroimaging at 3 years shows a small focus of gliosis at the site of cerebellar biopsy with resolution of inflammatory changes (e and f). Histological examination of cerebellar white matter biopsy: (g) H&E 20× demonstrating moderate parenchymal mixed inflammatory infiltrate of lymphocytes, plasma cells, and microglia with perivascular cuffing by lymphocytes without underlying vasculitis. Inset highlighting perivascular infiltrate of atypical lymphocytes. (h) CD3 20× highlighting both a parenchymal and perivascular T-cell infiltrate. (j) Luxol fast blue 20× highlighting evidence of diffuse white matter demyelination without perivascular accentuation of myelin loss. (i) CD8 20× and (k) CD4 20× – demonstrating a 2:1 ratio of CD4:CD8 T cells. (l) PD-1 20×: 30%–40% of the CD4-positive cells co-express PD-1 in keeping with Helper T cells. Bar = 100 μm.

On examination, there was reduced light touch sensation in the mandibular and maxillary distribution of the right trigeminal nerve with dysarthria, mild right upper limb weakness and dysmetria. Microcytosis (MCV 80.5 fL) with low ferritin (12 µg/L), lymphopenia (0.87 × 10^9^/L) and hypogammaglobulinaemia (IgG 5.2 g/L) were noted. Cerebrospinal fluid (CSF) analysis showed mildly elevated protein concentration (0.75 g/L), with normal white cell count (4/mm^3^) and the presence of unmatched (type 2) oligoclonal bands indicative of intrathecal IgG synthesis. CSF cytology and flow cytometry were non-diagnostic. Screening for infection including atypical organisms was negative although low-level Epstein–Barr virus (EBV) DNA was detected in her serum (EBV PCR 95 copies/mL; EBV DNA 1.98 log10 copies/mL). Brain magnetic resonance imaging (MRI) demonstrated a gadolinium-enhancing lesion in the right cerebellar peduncle with surrounding oedema and effacement of the fourth ventricle (Figure 1(a) and (d)).

Given clinical concern regarding CNS lymphoma, cerebellar biopsy was offered but was initially declined by the patient. Empirical treatment with pulsed methylprednisolone was initiated with clinical and radiological improvement (Figure 1(b)). However, relapse occurred on attempted corticosteroid wean; brain imaging demonstrated extension of the cerebellar lesion (Figure 1(c)). Biopsy of the right cerebellar peduncle lesion was subsequently performed. This demonstrated a florid active inflammatory and demyelinating process. The T-cell infiltrate was composed of CD4 and CD8 cells (approximately 2:1) and an estimated 30%–40% of the CD4 cells co-expressed programmed cell death protein 1 (PD-1), indicating T-cell activation (Figure 1(g)–(l)).

Subsequently, a virtual sub-panel of 194 genes associated with primary immunodeficiencies screened using Agilent ‘Focused Exome’ custom target enrichment system (SureSelectXT) and Next Generation Sequencing demonstrated that the patient (and her affected family members) were heterozygous for a novel, likely pathogenic frameshift deletion variant in *CLTA-4* exon 1:c.81dup p.(leu28Serfs*32). An additional novel heterozygous missense variant was identified in the autosomal recessive gene LRBA in exon 42: c.642T>C, p.(Phe2142Leu) but was considered of uncertain clinical significance given that only bi-allelic function loss variants have been linked with disease previously.

Pulsed corticosteroid therapy and oral taper was initiated with clinical benefit and sirolimus (an mTOR inhibitor which blocks the CD28 signalling pathway) was introduced as a steroid-sparing agent. Subsequently, the patient has been neurologically stable; reduced sensation in the maxillary nerve distribution on the right with a mild postural upper limb tremor but no eye signs or dysmetria. There has been resolution of neuroimaging changes and no recurrence of inflammatory CNS demyelination (Figure 1(e) and (f)).

## Discussion

Mendelian disorders of adaptive immunity have previously been reported to be associated with demyelination,^2^ and similarities reported in the aberrant peripheral B-cell responses of those with multiple sclerosis (MS) and immune dysregulation, polyendocrinopathy, enteropathy, X-linked (IPEX) syndrome are notable.^3^

In the largest case series reporting the clinical phenotype of CTLA-4 insufficiency, neurological involvement including autoimmune encephalitis or encephalomyelitis was reported in 28% of patients. This included three patients with cerebral perivascular lymphocytic infiltrates but, although demyelination was suspected clinically, this was not identified histologically.^4^ Here, we report CNS inflammatory demyelination and a novel, likely pathogenic *CTLA-4* frameshift deletion variant occurring in the context of primary immunodeficiency.

Increased CTLA-4 expression is protective in experimental allergic encephalomyelitis.^5^ Although *CTLA-4* polymorphisms are unlikely to have a significant effect on MS susceptibility,^6^ the *CTLA-4* polymorphism rs5742909 has been associated with reduced remyelination in MS^7^ and people with MS are known to have reduced expression of CTLA-4.^8^ Furthermore, therapeutic use of immune checkpoint inhibitors acting via the CTLA-4 pathway has been associated with clinically relevant demyelination.^1^ Disappointingly however, abatacept, a humanised CTLA4-IgG fusion protein, failed to demonstrate benefit in relapsing-remitting MS although note is made of the small study size.^9^

Given that lymphoma occurs with increased incidence in patients with autoimmunity including *CTLA-4* variants,^4^ there should be a low threshold to pursue a tissue diagnosis where there is evidence of neuroinflammation. This is particularly important given the delays in diagnosis which may be incurred following empirical corticosteroid treatment.^10^

This report of clinically relevant demyelination associated with an inflammatory T-cell infiltrate occurring in the context of a pathogenic *CTLA-4* variant, supports CTLA-4 as a regulator of T-cell activation and immune tolerance and is further evidence that perturbation of the CTLA-4 pathway can cause clinically relevant inflammatory demyelination. This has significant implication for the development of novel treatments for autoimmune conditions including MS but also emphasises the need for caution with clinical use of CTLA-4 immune checkpoint inhibitors, particularly in those with a history of immune-mediated demyelination. Consideration should be given to screening for *CTLA-4* variants in individuals with a history compatible with autoimmunity and CNS inflammatory disease, particularly following exclusion of lymphoma.
